# Personality Characteristics and Emotional Distress Among Chinese Pregnant Women: A Moderated Mediation Model

**DOI:** 10.3389/fpsyt.2021.645391

**Published:** 2021-11-18

**Authors:** Wenjiao Yang, Yanfei Hou, Yu Chen, Wenting Liu, Fan Fang, Julan Xiao, Jing Wang

**Affiliations:** School of Nursing, Southern Medical University, Guangzhou, China

**Keywords:** personality, depressive symptoms, anxiety symptoms, pregnant women, socioeconomic status (SES)

## Abstract

Previous studies have suggested that certain personality characteristics are associated with emotional distress during pregnancy. However, the underlying mechanism of this association is rarely understood. The current study investigated the links between personality and pregnant women's emotional distress (depressive and anxiety symptoms), tested the chain mediating effects of two resilience factors—social support and positive coping, and explored whether socioeconomic status (SES) could moderate the effects (including direct and/or indirect effects) of personality on their emotional distress. Results of a relatively large sample of pregnant women in China (*N* = 1157) showed positive associations for psychoticism and neuroticism with depressive and anxiety symptoms as well as negative associations for extraversion with depression and anxiety. After controlling for four important variables (the first pregnancy or not, having adverse pregnancy experience or not, being pregnant as planned or not, and number of weeks of pregnancy), social support and positive coping acted as chain mediators on the associations of personality with depressive symptoms as well as of personality with anxiety. Overall, the association of personality and depressive symptoms demonstrated invariance across socioeconomic status (SES). However, SES moderated the relationship between personality and anxiety. Specifically, the negative association of positive coping with anxiety symptoms was weaker for low SES women than for high SES ones. Results highlight the importance of social support and positive coping to decrease personality-related depressive and anxiety symptom among pregnant women. Furthermore, identifying other resilience factors that alleviate anxiety in women with low SES is urgently called for.

## Introduction

Emotional distress, including the symptoms of depression and anxiety, is very common among pregnant women. For instance, it is estimated that depression symptoms affect ~25% of pregnant women (1) and that anxiety symptoms affect 18.2–24.6% of them [*N* = 221,974; (2)]. The meta-analysis on perinatal depression among women residing in low-to-middle-income countries indicated that the pooled prevalence estimate of depression across 51 studies was 25.3% (3). The ratio of depressive and anxiety symptoms in the third gestational trimester has been reported to be as high as 73.5% and 58.5%, respectively (4). In China, prevalence rates of 15.04–22.57% for anxiety and 10.3–35.7% for depression have been reported (5, 6). Robust associations have been documented between emotional distress during pregnancy and maternal and newborn outcomes, such as postpartum emotional distress (7), and preterm birth and a reduction in breastfeeding initiation (8–10). Furthermore, emotional distress during pregnancy has far-reaching and adverse effects on an offspring's subsequent mental health (11, 12). Given the prolonged and adverse effects of emotional distress among pregnant women, it is urgently needed to investigate the associated factors and the underlying mechanisms to help clarify potential intervention objectives to prevent or decrease anxiety and depressive symptoms during pregnancy.

### Personality Characteristics and Depressive and Anxiety Symptoms Among Pregnant Women

In terms of the factors influencing depression and anxiety symptoms during pregnancy, negative experiences and environmental factors such as childhood abuse, general life stress, unplanned pregnancy, and being a single mother have received great attention in the literature (13–15). In addition to these factors, recent research has indicated the need to explore the relationship between personality characteristics and emotional distress in pregnancy (13, 16, 17).

“Personality characteristics” means a person's psychological and behavioral traits (18). Personality characteristics are relatively stable traits of an individual and include the tendency to respond to various stimuli in the same way (19). From the perspective of Eysenck (18), there are three independent personality characteristics: P (psychoticism; the trend to be aggressive, cold, tough-minded, antisocial, and insensitive to others), E (extraversion; the attitudes of interest, activation, and security for social interaction and the trend to have positive emotions such as enthusiasm, optimism, and happiness), and N (neuroticism; the trend to have strong and frequent negative emotions in the face of stressful situations).

Although the previous literature has shown that a person's personality characteristics might reflect individual differences in responses to environmental cues (20), only a few studies focus on the relationship between personality characteristics and emotional distress during pregnancy. For instance, a small longitudinal study (*N* = 96) suggested that neuroticism was significantly linked to anxiety levels during pregnancy (21). Bunevicius et al. (22) sampled 230 pregnant women and found that high neuroticism was an independent predictor of antenatal depressive disorders throughout pregnancy. A survey in China also reported that both high psychoticism and neuroticism personality characteristics are risk factors for depressive symptoms among Chinese pregnant women (17). A recent study in 85 pregnant women reported that psychoticism, neuroticism, and low extraversion were all cross-sectionally and longitudinally linked to depression symptoms (16). These limited empirical studies with relatively small samples indicated that the personality characteristics of pregnant women are closely associated with emotional distress and that pregnant women with high levels of neuroticism and psychoticism, as well as low levels of extraversion, might be more likely to experience depressive and/or anxiety symptoms. As personality characteristics may have a life-long effect on emotion, the effects of personality on emotion among pregnant women should be paid more attention.

Recently, research on postnatal women (*N* = 672) has begun to test the potential mechanisms of the associations between personality characteristics and postnatal depression. Postnatal anxiety is an important mediator in the relationship between the personality characteristic of extraversion and postnatal depression (23). Such research efforts, however, remain scarce. As the personality characteristic of a person is relatively stable and difficult to adjust, the study of intermediate variables (such as mediating and moderating variables) on the relationship between personality and emotion in pregnant women is urgently required, so as to help alleviate personality-related emotional distress through the development of interventions focused on the intermediate variables. Therefore, our study examined how personality characteristics correlated with depression and anxiety symptoms in a large sample of Chinese pregnant women, as well as the potential mediating and moderating mechanisms.

### Potential Chain Mediating Roles of Social Support and Coping Style

Previous research indicated that social support might act as a potential resilience resource for decreasing emotional distress of pregnant women (24–26). It is generally defined as material and spiritual supports and the exchange of material and spiritual resources between individuals, such that the individuals perceive that they are respected, loved, and cared for and have available assistance (27, 32). In interdependent cultures like in China, interpersonal harmony receives heavy emphasis (28); hence, the association between social support and depressive and anxiety symptoms among pregnant women has received a lot of recent attention. Results have consistently shown that low levels of subjective support and/or support utilization are important risk factors for depressive and anxiety symptoms in pregnant women (29–31). According to Xiao (32), social support is comprised of objective support, subjective support, and support utilization. Past research has shown that objective support was less determinative and valuable on emotional distress (33). Additionally, and linked with a tendency toward emotional distress, the N personality has been reported to be associated with low subjective support, and E is associated with increased subjective and utilization of support (16, 34, 35). We therefore expected that when pregnant women had higher levels of subjective and utilization of support, the extent to which they experience depression and anxiety decreases.

In addition to social support, coping style may also mediate the associations between individual characteristics and adaptive outcomes (36). Coping refers to the cognitive and behavioral styles adopted by an individual to manage the demands of stressors (37). Specifically, positive coping styles, such as planning, seeking advice, and engaging in activities, can relieve the impact of a stressor and thereby protect against emotional distress (38). Research with pregnant women has indicated that positive coping styles, including engagement in activities, making plans, and positive reframing, are associated with a significantly reduced occurrence of pregnancy complications (39). Negative coping styles, such as self-blame, denial, and substance abuse, correlated with high levels of depressive symptoms among pregnant women (40). Furthermore, there is a close link between individual personality characteristics and coping. For instance, when N is high and persistent, individuals are less likely to adopt positive coping styles (35). Therefore, it is rational to hypothesize that coping style could also play a similar role in assisting pregnant women prevent the adverse effects of negative personality characteristics. Hence, in the present study, we assumed that coping style may play a mediating role in the association between personality and emotional distress among pregnant women.

Furthermore, coping style, such as seeking advice from others, is positively associated with subjective and utilization of support. The buffering effects of social support on emotional distress are often mediated by the coping behaviors (41). For instance, research on pregnant women demonstrated that positive coping and subjective and utilization of support acted as chain mediators on the link of neuroticism and depressive symptoms after earthquake (42) as well as on the link of childhood abuse and emotional distress among them (43). Meanwhile, positive coping and subjective and utilization of support also acted as chain mediators between the relationship of childhood abuse and depressive symptoms on general adults (35). These might be that social support influences the choice of specific coping behaviors and the effectiveness of the behaviors used. Although there was no direct evidence related to the chain mediator effects of coping style and social support on anxiety in pregnant women, anxiety and depressive symptoms often occur simultaneously and have many common risk and protective factors. Therefore, it is reasonable to infer that positive coping and subjective and utilization of support may act as chain mediators on the relationship between personality characteristics and emotional distress (depressive and anxiety symptoms) among pregnant women.

### Moderating Role of Socioeconomic Status

The links of socioeconomic status (SES) and the well-being of pregnant women have received considerable attention from researchers (44, 45). Studies have examined several SES factors such as educational and economic levels in relation to antenatal anxiety and depression, but the results are equivocal. A survey of 5,398 pregnant women found that low educational level and low family income not only were directly and positively associated with anxiety and depression symptoms among them but also increased the disadvantageous effects of previous negative life events (46). Nevertheless, a survey on 583 pregnant women in Malawi found that those with more years of schooling tend to experience higher levels of anxiety and depression symptoms (47). Studies that have examined the emotional associations with low income have also reported contradictory results. While some studies found low income or financial difficulties to be related to severe emotional distress in pregnant women (48, 49), such associations have not been found in other studies. For instance, anxiety symptoms in pregnant women were not associated with their educational level (50). Also, there were no significant differences in the incomes of pregnant women with higher depressive symptoms compared with those with lower symptoms (51).

The above contradictory findings might be caused by individual differences among pregnant women such as personality characteristics, coping styles, and social support. Considering SES and personality, a recent meta-analysis and large online study (*N* = 2,183,377) found that high SES individuals experience fewer stressful events and more supportive environments, which results in more positive personality qualities (52). The individuals whose parents had a higher number of years of schooling were more likely to be open, extraverted, and emotionally stable (53). Another study indicated that positive coping style was significantly and positively correlated with SES, while negative coping was not (54).

Furthermore, according to the stress-buffering model, the adverse effects of stressors on the development of individuals would be weaker for those with plenty of resources (55). The reasons may be that the resources can alleviate the deleterious impacts of stressors. Usually, individuals with high SES perceive less stress (56), adopt more positive coping styles (57), and have more social support (52). Therefore, it was rational to assume that SES could moderate the direct and/or indirect pathways from personality to both depressive and anxiety symptoms. Specifically, compared with pregnant women with a low level of SES, the negative associations of personality characteristics and emotional distress might be smaller for women with a high level of SES.

### Present Study

Although there is some research available on the relationships between personality and depression and anxiety in pregnant women, most studies have been conducted in small samples and there has been little attempt to assess the potential mediating roles of social support and coping styles. Hence, to fill these gaps in the literature, our main aim in the present study was to investigate how and under what conditions personality would be linked with emotional distress in a large sample of Chinese pregnant women. Specifically, the current study sought to specify the association between personality and emotional distress by considering social support and coping style as potential mediators and SES as a potential moderator. Based on the previous literature, we assumed that neuroticism and psychoticism would positively correlate, and extraversion would negatively correlate, with pregnant women's depressive and anxiety symptoms (H1: the relationship hypothesis). Furthermore, we expected that higher neuroticism and psychoticism, and lower extraversion, would be associated with higher levels of emotional distress through lower levels of social support and positive coping and that social support and positive coping acted as chain mediators (H2: the mediating hypothesis). Finally, we also assumed that SES would have a moderating role in the direct and/or indirect links between personality and emotional distress (H3: the moderating hypothesis).

## Materials and Methods

### Participants

Potential participants were pregnant females visiting the obstetrics and gynecology wards at six general hospitals in six cities (*N* = 314 in Guangzhou city, *N* = 166 in Foshan city, *N* = 153 in Zhaoqing city, *N* = 210 in Huizhou city, *N* = 177 in Shenzhen city, and *N* = 137 in Qingyuan city) of Guangdong Province, China, for a conventional examination between March 2018 and July 2019. During the period of waiting for their appointments, they were asked by investigators to participate in the present study. The inclusion criteria included being more than 18 years old and being in any period of pregnancy. A total of 1,250 women gave their consent to be recruited in this study and accomplished a questionnaire in a quiet office which took around 15 min. However, 36 participants withdrew before they completed the questionnaires. Among the 1,214 questionnaires collected, 39 in-completed surveys and 18 questionnaires with implausible answers were excluded from analysis, leaving 1,157 valid questionnaires for the final analysis (reflecting a valid response rate of 95.30%). There was no significant difference in demographic data between included women and excluded women.

The mean age of the pregnant women was 28.91 years (*SD* = 4.57, range 18–44 years). Most participants were younger than 35 years (*n* = 1,020, 88.2%). The educational levels were as follows: 379 (32.8%) had completed more than 16 years of education or a bachelor's degree, 404 (34.9%) had completed 14–15 years of education or 2–3 years of college studies, 209 (18.1%) had completed 12 years of education or senior middle school studies, and finally, 165 (14.3%) had not completed either 9 years of education or junior middle school studies. Meanwhile, the education levels of the husbands of participants were as follows: 458 (39.6%) had completed more than 16 years of education or a bachelor's degree, 372 (32.2%) had completed 14–15 years of education or 2–3 years of college studies, 191 (16.5%) had completed 12 years of education or senior middle school studies, and finally, 136 (11.8%) had not completed either 9 years of education or junior middle school studies. Furthermore, the monthly household incomes were reported as follows: 392 (33.9%) had over 10,000 yuan, 470 (40.6%) had 5,000–9,999 yuan, 250 (21.6%) had 3,000–4,999 yuan, and 45 (3.9%) had <2,999 yuan.

### Procedure

The procedure was approved by the review board (number 71874075) at Southern Medical University in China before the start of the survey and was in accordance with the ethical standards of the responsible committee on human research of our institution and with the Helsinki Declaration. We have also adhered to standard bio-security and institutional safety procedures. In the preparation period, all investigators have been trained to ensure that they use the same instructions and provide assistance or clarification to participants if needed, so as to guarantee the quality of data collection. Meanwhile, we communicated with the persons-in-charge of the above six hospitals and were approved to conduct research in their institution. Furthermore, before participating in this study, all participants had been informed of the aim of the current study and the nature of voluntary participation. Subjects who agreed to take part in were directed to fill out the questionnaire anonymously in the waiting area.

### Measures

The socio-demographic features, personality characteristics, social supports, coping styles, and depressive and anxiety information were gathered using questionnaires. The gathered socio-demographic feature covered age, education level of the pregnant women and their husbands, and monthly household income.

#### Personality Characteristics

The Chinese version of the Eysenck Personality Questionnaire-Revised Short Form [EPQ-RS; (58)] was used to measure the personality characteristics of the participants. The EPQ-RS was translated from the English-version questionnaire (59). It is comprised of 48 items and assesses the three subscales of P, E, and N, and L was additionally used to measure dissimulation and provide a validity scale. Responses were scored 0 or 1, and some items were reverse-scored. Therefore, the scores of each subscale (P, E, N) ranged from 0 to 12, with higher scores demonstrating higher levels of each personality characteristic. The EPQ-RS has been widely used in China and has acceptable reliability and validity (60, 61). The Cronbach's α coefficient for the subscales were 0.72 (P), 0.79 (N), and 0.76 (E) in this study.

#### Perceived and Used Social Support

Social support was measured by the Social Support Rating Scale [SSRS; (62)]. The SSRS was specially designed for utilization in the Chinese context. It includes 10 items with three dimensions: objective support (three items), subjective support (fur items), and support utilization (three items). As objective support had less determinative and valuable role on emotional distress (33), it was not included in the present study. The scores of subjective support ranged from 8 to 32, and support utilization scores ranged from 3 to 12. The higher the scores, the higher the level of social support. The SSRS has been widely used among pregnant women in China and has high reliability and validity (35). The Cronbach's α was 0.73 for subjective support and 0.71 for support utilization in the present study.

#### Positive Coping

The Chinese version of the Simplified Coping Style Questionnaire [SCSQ; (63)] was employed to measure coping tendencies of pregnant women. The SCSQ was modified by Xie according to the Chinese context, on the basis of the Ways of Coping Questionnaire (64). It includes 20 items and two dimensions: positive coping style (12 items) and negative coping style (8 items). Positive coping style emphasizes active coping characteristics, such as engagement in activities, planning, and talking to others, while negative coping style emphasizes the features of passive coping, such as substance use, relying on others, and fantasy. Agreement with each statement is assessed on a three-point Likert-type scale ranging from 0 (none) to 3 (always). The total scores for positive coping and negative coping were calculated separately, with a higher score representing a more frequent utilization of the corresponding coping style. The SCSQ has reported to have good reliability among Chinese pregnant women (65). The Cronbach's alpha was 0.89 for positive coping and 0.70 for negative coping in the present study.

#### Depressive Symptoms

The depressive symptom over the past week was assessed by the Chinese version of the Edinburgh Postnatal Depression Scale [EPDS; (66)], which was translated from the English version (67). The EPDS contains 10 self-rating items. Agreement with each statement is measured on a four-point Likert-type scale ranging from 0 (none) to 3 (yes, most of the time). Add all the answers together for a total score, and the higher the score, the higher the level of depressive symptoms. It showed good validity and reliability among Chinese pregnant women (68). In the present study, the Cronbach's α was 0.75.

#### Anxiety Symptoms

The anxiety symptom over the past week was measured through the Chinese version of the Self-Rating Anxiety scale [SAS; (69)]. The SAS was modified from the English-version scale (70). It is a 20-item self-rating instrument, and responses to the items were scored from 1 (none) to 4 (most or all of the time). All the responses were summed up to generate a total score (ranging from 20 to 80). The higher the score, the higher the level of anxiety symptoms. The SAS has been widely adopted among Chinese pregnant samples and has high validity and reliability (5, 71). In this study, the Cronbach's alpha was 0.76.

#### SES

The SES factors in the current study included education degree of pregnant women, education degree of the husband, and monthly household income (46, 72). Pregnant women were asked to report the education level of themselves and their husbands from 1 to 4 (1 = 9 years of education or below junior middle-school studies, 2 = 12 years of or senior middle-school studies, 3 = 14–15 years of education or 2–3-year college studies, 4 = 16 years of education or over bachelor's degree). Women also reported their monthly household income as either >10,000 yuan, 5,000–9,999 yuan, 3,000–4,999 yuan, or <2,999 yuan, scored as 1–4. The responses to these three questions were added up to generate the total score. Then, according to the median segmentation, the total scores of SES were recorded as a dichotomous variable [high SES group and low SES group; (72)].

#### Controlling Variables

Controlling variables included the first pregnancy or not, having adverse pregnancy experience or not, being pregnant as planned or not, and number of weeks of pregnancy, which were reported to influence the emotional distress of pregnant women (73, 74). The former three variables were dummy coded (1 = *ever having pregnancy experience*, 0 = *in the first pregnancy*; 1 = *having adverse pregnancy experience*, 0 = *not having adverse pregnancy experience*; 1 = *unplanned pregnancy*, 0 = *planned pregnancy*), and the number of weeks of pregnancy was a continuous variable. Among the sample in the present study, 688 (59.5%) reported to have pregnancy experience ever and 469 women (40.5%) were in their first pregnancy. Ninety-one women (7.9%) reported to have adverse pregnancy experience, while 1,066 (92.1%) had not. Three hundred one women (26.0) were pregnant as planned, and 856 (74.0%) had unintended pregnancies. The mean pregnancy duration was 27.15 weeks (*SD* = 9.50, range 6–40 weeks).

## Statistical Analysis

Statistical analyses were performed using SPSS 23.0 and AMOS 23.0. Firstly, the data were screened for outliers and to measure the linearity and normality. An absolute skew value below 2 is considered to be within the acceptable range of normality (75). Then, we performed descriptive statistics of the study variables. Furthermore, we performed Pearson correlation analysis to examine the relationships among continuous variables and point-biserial correlation to test the associations between dichotomous variables and continuous variables.

Later, structural equation modeling (SEM) with AMOS 23.0 was adopted to test the potential chain mediating effect assumptions. To decrease collinearity, we mean-centered all continuous variables in the present study. The maximum likelihood estimation method was selected and used for the SEM. According to Wu (76), the overall model fit was assessed using several fit indexes. Specifically, we compared and selected models based on the ratio of χ^2^ to degrees of freedom (χ^2^/df) and on these fit indexes: the root mean square error of approximation (RMSEA), the incremental fit index (IFI), the normed fit index (NFI), and the Bentler comparative fit index (CFI). For χ^2^/df and RMSEA, values below 5 and 0.08 indicate an acceptable model fit separately. For IFI, NFI, and CFI, values over 0.90 indicate a good model fit. In the chain mediation analysis, bootstrapping was used to obtain confidence intervals (CIs) based on 10,000 samples (77).

Finally, we tested SES as the possible moderator of the chain mediating model using multigroup analysis. The SES was recorded into dichotomous variable by means of median splits. Based on Wu (78), the tests of differences in the SEM framework were as follows. Firstly, we tested the hypothesized structure without constraining any parameters in both groups at the same time (named baseline model or unconstrained model). If the baseline model was sufficiently fit, we forced some of the parameters (i.e., measurement residuals, measurement weights, and structural residuals) to be equal for the two groups (called constrained model) and compared the constrained model to the baseline model. If the statistical fit of the constrained model showed a significantly worse solution (the significant increase in χ^2^ value) than that of the unconstrained model, this indicated that at least one parameter was different between the groups. If multiple models fitted the data model, the final model was selected based on the indexes of Δχ^2^, AIC, and expected cross-validation index (ECVI).

In both the chain mediating effect test and the moderating test, four important variables (the first pregnancy or not, having adverse pregnancy experience or not, being pregnant as planned or not, and number of weeks of pregnancy) which might influence emotional distress of pregnant women (73, 74) were set as control variables.

## Results

### Descriptive Statistics and Univariate Correlations

Descriptive statistics for the study variables are displayed in Table 1. The skewness values of the study variables were all below 2, representing that they were not substantially skewed. Pregnant women's extraversion was positively related to positive coping and social support, while psychoticism and neuroticism were negatively related to these three variables. As expected, positive coping and social support were all negatively correlated with depressive and anxiety symptoms. Pregnant women's extraversion had a negative association with depression and anxiety symptoms, while psychoticism and neuroticism had a positive relationship. The degree of these correlations ranged from mild to moderate. However, negative coping did not significantly correlate with extraversion, subjective support, or support utilization. Therefore, negative coping was excluded in the following mediating and moderation analysis.

**Table 1:** Univariate correlations of study variables (*N* = 1157).

	**Skewness**	**1**	**2**	**3**	**4**	**5**	**6**	**7**	**8**	**9**
1. Psychoticism	1.07	1								
2. Extraversion	−0.66	0.040	1							
3. Neuroticism	0.55	0.430^**^	−0.080^**^	1						
4. Positive coping	−0.14	−0.193^**^	0.324^**^	−0.222^**^	1					
5. Negative coping	0.55	0.164^**^	−0.031	0.160^**^	0.312^**^	1				
6. Subjective support	−0.62	−0.132^**^	0.183^**^	−0.283^**^	0.172^**^	−0.045	1			
7. Support utilization	0.10	−0.077^**^	0.209^**^	−0.194^**^	0.248^**^	0.042	0.342^**^	1		
8. Depressive symptoms	1.00	0.296^**^	−0.259^**^	0.422^**^	−0.320^**^	0.19^**^	−0.252^**^	−0.223^**^	1	
9. Anxiety symptoms	0.72	0.325^**^	−0.196^**^	0.466^**^	−0.253^**^	0.219^**^	−0.177^**^	−0.115^**^	0.552^**^	1

** *p < 0.01*.

### Chain Mediating Effects of Social Support and Positive Coping

We first checked the chain mediating effects of social support and positive coping in the relationship between personality characteristics and depressive symptoms. In order to induce the collinearity, we mean-centered all variables. After controlling for the first pregnancy or not, having adverse pregnancy experience or not, being pregnant as planned or not, and number of weeks of pregnancy, the associations between psychoticism and social support as well as between neuroticism and positive coping were not significant (*p*s > 0.05). Therefore, we deleted these two links from the mediating model. Then, the overall fitting index showed that the fitting between the model and the data were acceptable (χ^2^/df = 4.206; RMSEA = 0.053, 90% CI: 0.043, 0.062; CFI = 0.927, NFI = 0.909, IFI = 0.929). Furthermore, results indicated that neuroticism had a negative association with social support, while extraversion had a significant positive association. Pregnant women's extraversion was positively related to positive coping, while psychoticism was negatively related. Pregnant women's social support and positive coping correlated negatively with depressive symptoms. Meanwhile, social support was positively correlated with their positive coping.

**Figure 1:**
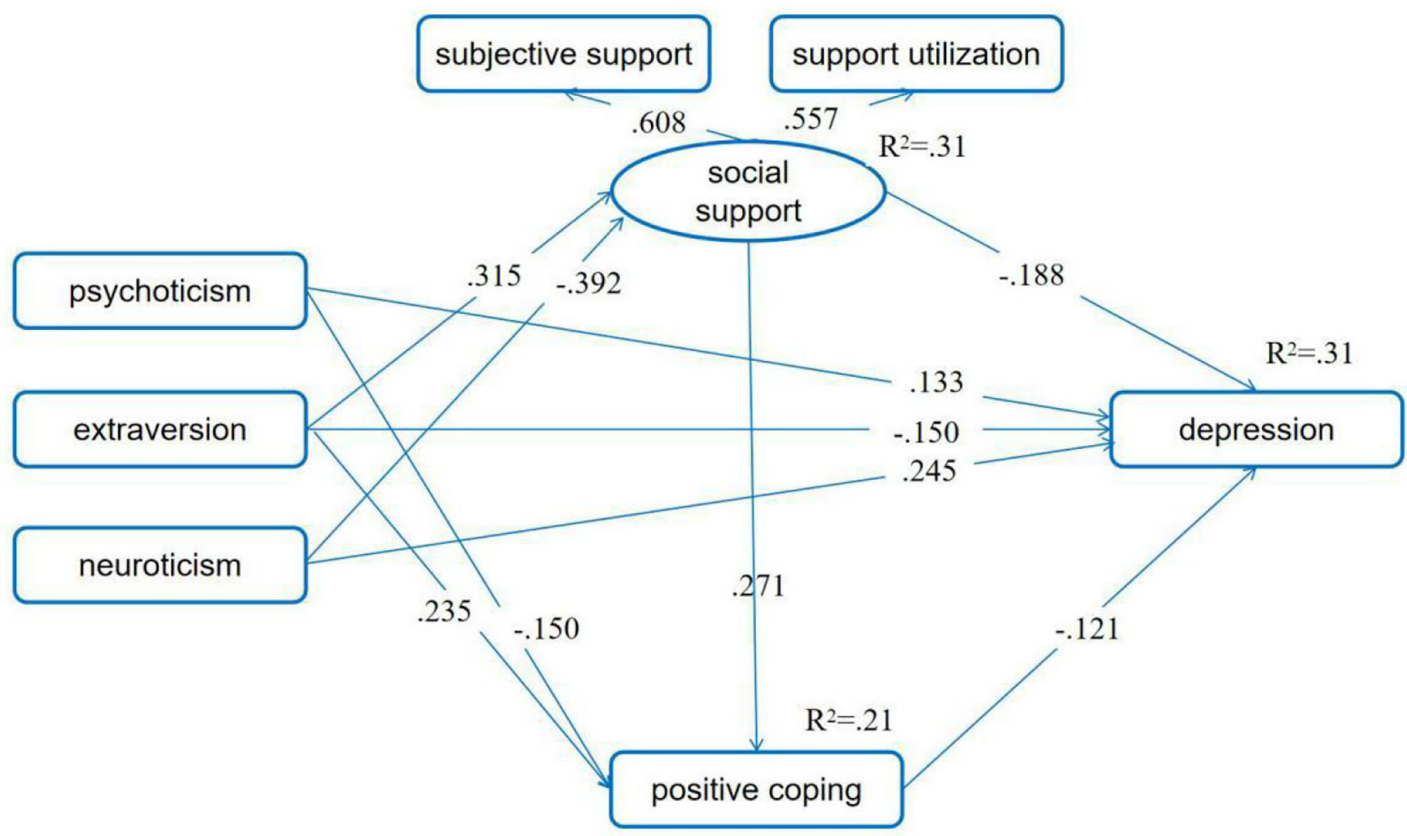
Positive coping and social support as chain mediators between personality and depressive symptoms. Standardized path coefficients are presented in the model (*p*s < 0.05). The first pregnancy or not, having adverse pregnancy experience or not, being pregnant as planned or not, and number of weeks of pregnancy were set as control variables.

The direct paths from psychoticism, extraversion, and neuroticism to depression were all significant (standardized direct effect = 0.133, 90% BCIs: 0.078, 0.191; −0.150, 90% BCIs: −0.204, −0.099; 0.245, 90% BCIs: 0.182, 0.308). Furthermore, all three personality characteristics correlated significantly and indirectly with depressive symptoms (standardized indirect effect = 0.018, 90% BCIs: 0.009, 0.030; −0.098, 90% BCIs: −0.129, −0.071; 0.086, 90% BCIs: 0.053, 0.130). Furthermore, extraversion and neuroticism correlated significantly and indirectly with positive coping (standardized indirect effect = 0.085, 90% BCIs: 0.053, 0.122; −0.106, 90% BCIs: −0.143, −0.074). Social support also correlated significantly and indirectly with depressive symptoms (standardized indirect effect = −0.033, 90% BCIs: −0.053, −0.017). These indicated that social support and positive coping acted as chain mediators among the relationship of personality and depressive symptoms (Figure 1).

We then checked the mediating effect of social support and positive coping in the relationship between personality and anxiety symptoms. After controlling for the first pregnancy or not, having adverse pregnancy experience or not, being pregnant as planned or not, and number of weeks of pregnancy, the results indicated that the associations between social support and anxiety symptoms, between psychoticism and social support, and between neuroticism and positive coping were not significant (*p* > 0.05). Therefore, we delete these three links. Then, the fitting indexes indicated that the fitting between the model and the data was acceptable (χ^2^/df = 4.106; RMSEA = 0.052, 90% CI = 0.043, 0.062; CFI = 0.926, NFI = 0.907, IFI = 0.928). Meanwhile, the results showed that neuroticism had a negative association with social support, while extraversion had a significant positive association. Pregnant women's extraversion was positively related to positive coping, while psychoticism was negatively related. Pregnant women's positive coping correlated negatively with depressive symptoms. Meanwhile, social support was positively correlated with their positive coping.

**Figure 2:**
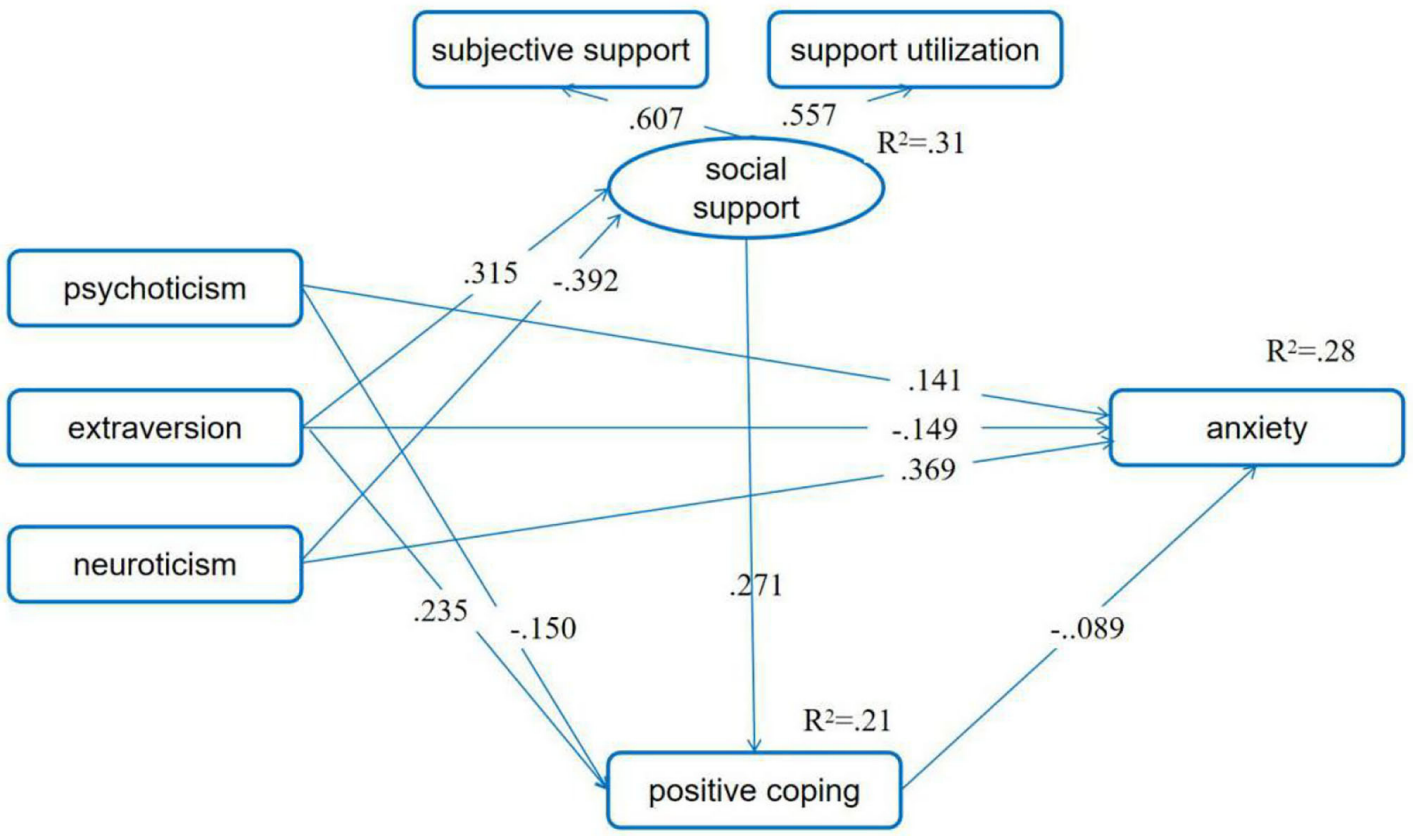
Positive coping as mediator between personality and anxiety symptoms. Standardized path coefficients are presented in the model (*p*s < 0.05). The first pregnancy or not, having adverse pregnancy experience or not, being pregnant as planned or not, and number of weeks of pregnancy were set as control variables.

The direct paths from psychoticism, extraversion, and neuroticism to depression were all significant (standardized direct effect = 0.146, 90% BCIs: 0.094, 0.200; −0.149, 90% BCIs: −0.200, −0.098; 0.369, 90% BCIs: 0.320, 0.414). Furthermore, all three personality characteristics correlated significantly and indirectly with depressive symptoms (standardized indirect effect = 0.013, 90% BCIs: 0.006, 0.023; −0.029, 90% BCIs: −0.046, −0.012; 0.009, 90% BCIs: 0.004, 0.017). Furthermore, extraversion and neuroticism correlated significantly and indirectly with positive coping (standardized indirect effect = 0.085, 90% BCIs: 0.057, 0.122; −0.106, 90% BCIs: −0.143, −0.073). Social support also correlated significantly and indirectly with depressive symptoms (standardized indirect effect = −0.024, 90% BCIs: −0.041, −0.011). These suggested that social support and positive coping acted as chain mediators on the relationship of personality and anxiety symptoms (Figure 2).

### Moderating Effect of SES: Multigroup Analysis

In order to examine whether the associations between personality and emotional distress found in the whole participant samples are also applicable to the high SES and low SES subgroups, we conducted the multigroup analysis. We also controlled for the first pregnancy or not, having adverse pregnancy experience or not, being pregnant as planned or not, and number of weeks of pregnancy in the following multigroup analysis.

**Figure 3:**
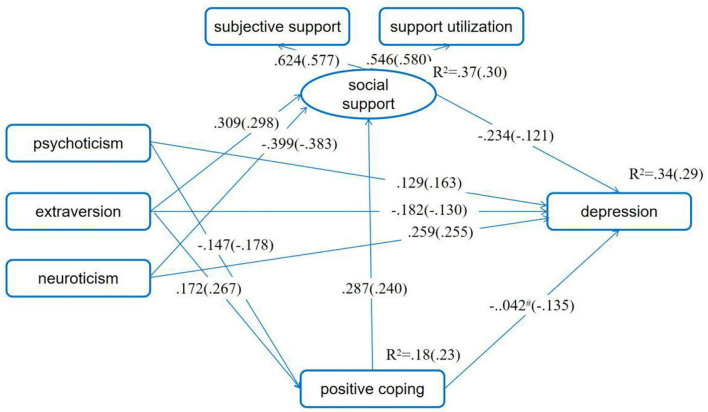
SES as moderator in the chain mediating model of positive coping and social support in the association between personality and depressive symptoms. Standardized path coefficients before parentheses were for the low SES group, and those in the parentheses were for the high SES group. ^#^*p* > 0.05, while the *p*-values for other standardized path coefficient were all <0.05. The first pregnancy or not, having adverse pregnancy experience or not, being pregnant as planned or not, and number of weeks of pregnancy were set as control variables.

For the moderating effect of SES in the relationship between personality and depressive symptoms, the unconstrained model and the measurement weight model fit the data well. Although the values of CFI, NFI, and IFI for the structural covariance model and the measurement residual model were < 0.90, the other indexes were good. These suggested that the hypothesized four models were acceptable. We selected the measurement weight model as the final model according to the values of χ^2^, AIC, and ECVI. Then, we compared path coefficients for women with low SES and those with high SES one by one and found that there were no significant differences between all the path coefficients for the two groups (*p*s > 0.05). Results of these indicated that the chain meditating model of social support and positive coping in the association between personality and depressive symptoms demonstrated invariance across SES (Figure 3; Table 2).

**Table 2:** Goodness-of-fit indices for model comparisons in moderation analysis on the mediation model.

**Model**	**χ^**2**^**	***df***	**χ^**2**^/df**	**Δχ^**2**^**	**Δdf**	***p*-value for Δχ^**2**^**	**RMSEA (90% CI)**	**CFI**	**NFI**	**IFI**	**AIC**	**ECVI**
Threshold for acceptable fit			<5			≥0.05 (significant level)	<0.08	≥0.90	≥0.90	≥0.90		
Depressive symptoms											
Unconstrained	145.676	58	2.512				0.036 (0.029,0.044)	0.932	0.905	0.934	293.676	0.254
Measurement weights	147.742	61	2.422	2.066	3	0.559	0.035 (0.028,0.042)	0.932	0.904	0.935	289.742	0.251
Structural covariances	242.777	90	2.689	97.100	32	<0.001	0.038 (0.033,0.044)	0.881	0.856	0.883	326.777	0.283
Measurement residuals	255.365	95	2.688	109.689	37	<0.001	0.038 (0.033,0.044)	0.875	0.847	0.876	329.365	0.285
Anxiety symptoms											
Unconstrained	151.528	60	2.525				0.036 (0.029,0.044)	0.929	0.908	0.932	295.528	0.256
Measurement weights	152.108	62	2.453	0.581	2	0.748	0.035 (0.028,0.043)	0.930	0.905	0.933	292.108	0.253
Structural covariances	259.724	91	2.854	108.196	31	<0.001	0.040 (0.034,0.046)	0.869	0.835	0.871	341.724	0.296
Measurement residuals	264.007	96	2.750	112.479	36	<0.001	0.039 (0.033,0.045)	0.870	0.832	0.871	336.007	0.291

*χ^2^, chi-square; df, degree of freedom; χ^2^/df, the chi-squared/freedom ratio; RMSEA, root mean square error of approximation; CFI, comparative fit index; NFI, normed fit index; IFI, incremental fit index; AIC, Akaike information criteria; ECVI, expected cross-validation index. The chi-square deference (Δχ^2^) tests are compared with the unconstrained model (baseline model)*.

**Figure 4:**
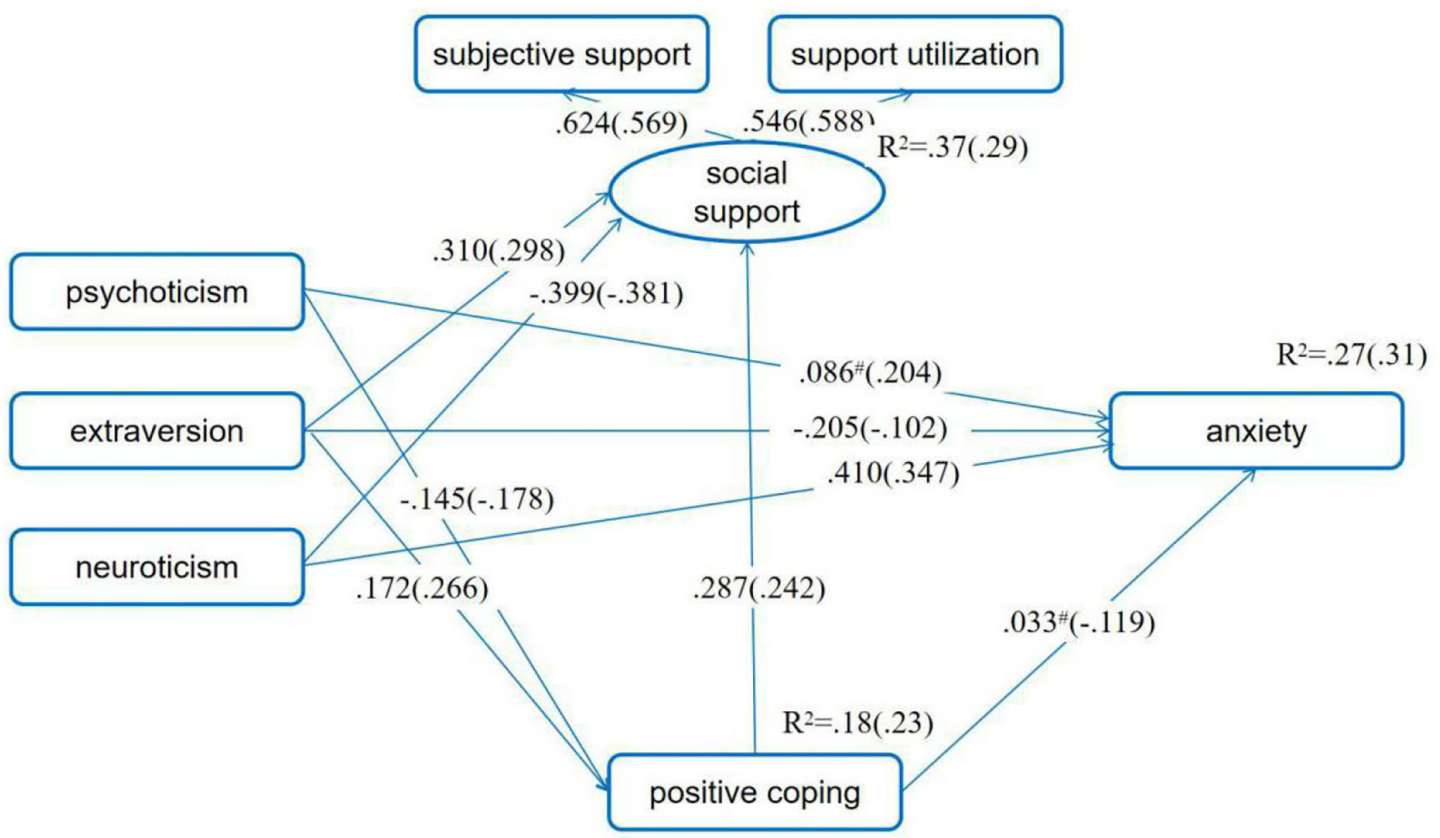
SES as moderator in the mediating model of positive coping in the association between personality and anxiety symptoms. Standardized path coefficients before parentheses were for the low SES group, and those in the parentheses were for the high SES group. ^#^*p* > 0.05, while the *p*-values for other standardized path coefficient were all <0.05. The first pregnancy or not, having adverse pregnancy experience or not, being pregnant as planned or not, and number of weeks of pregnancy were set as control variables.

Concerning the proposed moderating effect of SES in the mediating model of the relationship between personality and anxiety, results showed that both the unconstrained model and the measurement weight model fit the data well. Although the values of CFI, NFI, and IFI for the structural covariance model and the measurement residual model were below 0.90, the other indexes were good. These indicated that the hypothesized models were acceptable. We also selected the measurement weight model as the final model based on the values of χ^2^, AIC, and ECVI. Through comparing path coefficients for women with low and high SES, we found that the relationship between positive coping and anxiety was not significant for low SES women (standardized direct effect = 0.033, 90% BCIs: −0.061, 0.133, *p* = 0.455) but was significant for high SES women (standardized direct effect = −0.119, 90% BCIs: −0.191, −0.043, *p* < 0.001), and the difference in the pathways was significant (*t* = −2.652, *p* < 0.01). These suggested that the negative association between positive coping and anxiety symptoms was weaker for pregnant women with low SES than for those with high SES (Figure 4; Table 2).

## Discussion

In line with the relationship hypothesis (H1), the present study indicated that personality characteristics correlated moderately or mildly with depressive and anxiety symptoms among Chinese pregnant women. Also consistent with the mediating hypothesis (H2), after controlling for four important variables (the first pregnancy or not, having adverse pregnancy experience or not, being pregnant as planned or not, and number of weeks of pregnancy), social support and positive coping acted as chain mediators on the link between personality and depressive symptoms as well as between personality and anxiety. As predicted (H3: the moderating hypothesis), SES played a moderating role in the chain mediating model of personality and anxiety symptoms. Specifically, the negative link between positive coping and anxiety symptoms was weaker for low SES women than for high SES ones. However, inconsistent with the assumption, the chain mediating effects of social support and positive coping on the link of personality and depressive symptoms demonstrated invariance across SES.

In light of pregnant women's emotional distress becoming more common (3, 79), our findings indicate that positive coping style and subjective and utilization of support are important mediators on the relationship between personality and emotion among Chinese pregnant women. Furthermore, the mediating effect of positive coping was non-existent in the anxiety model among low SES pregnant women. Therefore, more attention should be paid to the anxiety symptom experienced by this specific group.

In accordance with past research (16, 22), there were positive associations for psychoticism and neuroticism with emotional distresses as well as negative associations for extraversion with emotional distresses among pregnant women. These indicated that when pregnant women had high levels of psychoticism or neuroticism, they were more likely to become depressed and anxious. However, if the pregnant women had high levels of extraversion, they tended to experience less depression and anxiety symptoms. These above results suggest that improving the extroversion and reducing psychoticism and neuroticism are helpful to alleviate the anxiety and depressive symptoms of pregnant women. Furthermore, as personality characteristics are formed in the early years of life and are relatively stable in adulthood (80), it is relatively difficult to adjust them to substantially decrease the likelihood of emotional distress among adult pregnant women. Therefore, it is critical to study the mediators and moderators in the relationships between personality and emotional distress and to provide empirical evidence for the intervention of depressive and anxiety symptoms during pregnancy.

Although empirical findings link personality characteristics and depression symptoms in pregnancy, little attention has been paid to how to explain these relationships in the literature. In the present study, we assessed whether social support and positive coping may explain these associations. Taken together, we found significant chain mediating effects of social support and positive coping on the associations of personality characteristics and depressive symptoms as well as of personality and anxiety. Specifically, when pregnant women were lower in psychoticism and neuroticism, and higher in extraversion, they were more likely to have high levels of social support and/or positive coping, which correlated negatively with depression and anxiety symptoms. This is consistent with past research (42, 43). Results of these are also in line with the psychosocial theory (81). Notably, in this chain mediating model, the psychoticism was not directly related to social support and neuroticism was not directly related to positive coping.

More importantly, to our knowledge, the present study was the first to explore whether SES could moderate the mediated effects of personality characteristics on emotional distress through social support and positive coping. Inconsistent with the assumption, we did not find the moderating effect of SES on the association of personality with depressive symptoms among pregnant women. In other words, the relationships of personality characteristics to emotional distress through social support and positive coping did not vary with SES. This expands the past literature and supports the important roles of both social support and positive coping on alleviating the personality-related depressive symptoms for pregnant women.

Findings from the present study indicated that the negative link between positive coping and anxiety symptoms was weaker for low SES women than for high SES ones. These are consistent with the stress-buffering model (55). In comparison of high SES individuals, those with low SES typically had limited resources [such as receiving less meritorious information and advice as well as obtaining less frequent technological and material assistance; (82, 83)], which might be insufficient to promote the effect of positive coping on personality-influenced emotional distress. Furthermore, individuals with low SES experience more stress than those with high SES backgrounds (84) and stress is linked with emotional distress and other negative outcomes (85). This may mean that low SES pregnant women may suffer more stressful events and have fewer resources. Furthermore, the unexpected null findings in pregnant women with low SES might imply that other unmeasured resilience factors for anxiety symptom associated with the low SES, such as sense of control and life-meaning, may have played more contributory roles than the positive coping.

Furthermore, these results might be explained by the differences in personality characteristics between women with high SES and women with low SES. Women with low SES typically had higher levels of neuroticism, such as lower emotional stability, than high SES women (52). Therefore, for pregnant women with the same level of positive coping, those with low SES would be less likely to relieve the negative relationships of their psychoticism and neuroticism and anxiety, as well as promote the positive association between their extraversion and anxiety symptom. As the latest study stated, pregnancy-specific anxiety, the condition marked by worries, concerns and fears about pregnancy, childbirth, the health of the infant, and future parenting, is an under-recognized area and deserves more attention (86). Findings in the present study also suggest that more research into the associations between personality characteristics and anxiety symptom in low SES pregnant women is urgently called for, especially on the alleviation of the links between negative personality characteristics (psychoticism and neuroticism) and anxiety, and the enhancement of the positive links between positive personality characteristics (extraversion) and anxiety symptom.

### Limitations and Conclusions

The current study also has some limitations. First, we relied on self-reports to measure the main constructs. The observations and holistic assessments of various aspects of life among pregnant women are needed to confirm and enhance the current findings. Further studies could also benefit from qualitative interviews to investigate correlations between personality and emotional distress in a more nuanced approach. Second, as with past cross-sectional surveys, the results of the present study could not offer time series or clear causal conclusion. Thus, longitudinal studies are necessary to evaluate potential etiological relationships between personality, coping, support, and emotional distress among pregnant women. Third, the psychiatric assessment was missing in the present study. In future studies, psychiatric assessments should be included to assess and exclude subjects with psychiatric disorders, as psychiatric disorders may affect self-reporting of personality traits. Furthermore, it should be noted that the pregnant women in this study were all from Guangdong province, a relatively developed province in China. Therefore, the moderating role of SES requires to be assessed among pregnant women in relatively poor provinces in China. Finally, the associations between personality and pregnant women's depressive and anxiety symptoms in the present study were relatively mild. However, since personality characteristics have a prolonged effect on each individual's emotion, the mild links found may have a crucial practical impact on pregnant women over time.

Despite these limitations, this study expands the literature on the direct associations of personality characteristics and emotional distress among pregnant women in China. Wu also found that personality characteristics correlate indirectly with emotion through the buffering effect of social support and positive coping. That is, social support and positive coping can serve as beneficial factors on the relationship between personality and emotional distress (depressive and anxiety symptoms) among pregnant women. Furthermore, the chain mediating effects of social support and positive coping on the link of personality characteristics and anxiety varied according to SES. Specifically, for pregnant women with low SES, studies on the impact of other mediators, such as sense of control and life-meaning, on the link between personality characteristics and anxiety symptom are needed. As such, this study highlights the need to comprehensively consider both social support and coping style to prevent depressive and anxiety symptoms among Chinese pregnant women.

The present study has theoretical and practical implications. It contributes to better understandings of how pregnant women's personality characteristics are related to their emotional distress and might increase the possibility of a positive pregnancy experience. This study has also helped to better understand the buffering effects of social support and positive coping in the personality–emotional distress associations. Furthermore, these results are crucial for those who play an important role in women's pregnancy experiences, such as doctors, nurses, and psychotherapists. The results of the current study could also be used to develop clinical intervention and management programs for emotional distress in pregnant women. For instance, for pregnant women with high levels of depressive and anxiety symptoms, training in coping styles and perceiving and using support might help alleviate symptoms of pregnant women.

## Data Availability Statement

The datasets used and/or analyzed during the current study are available from the corresponding author on reasonable request.

## Ethics Statement

The study was approved by the Ethical Review Board of Institute, Southern Medical University. The participants provided their written informed consent to take part in this study.

## Author Contributions

YC: study design. WY, FF, JX, and JW: data collection. YH: data analysis. YH, WY, and YC: manuscript preparation. All authors contributed to the article and approved the submitted version.

## Funding

This work was supported by the National Natural Science Foundation of China [No. 71874075] and Humanities and Social Sciences of Ministry of Education [No. 18YJAZH008].

## Conflict of Interest

The authors declare that the research was conducted in the absence of any commercial or financial relationships that could be construed as a potential conflict of interest.

## Publisher's Note

All claims expressed in this article are solely those of the authors and do not necessarily represent those of their affiliated organizations, or those of the publisher, the editors and the reviewers. Any product that may be evaluated in this article, or claim that may be made by its manufacturer, is not guaranteed or endorsed by the publisher.
